# Supplementary material for: Correlation of Autophagosome Formation with Degradation and Endocytosis Arabidopsis Regulator of G-Protein Signaling (RGS1) through ATG8a
Source: Int J Mol Sci. 2019 Aug 27;20(17):4190. doi: 10.3390/ijms20174190 (PMC6747245; doi:10.3390/ijms20174190)
Supplement: Supplementary file 1 [file ijms-20-04190-s001.zip › manuscript supplementary.pdf]

International Journal of
Molecular Sciences

MDPI

Supplementary data

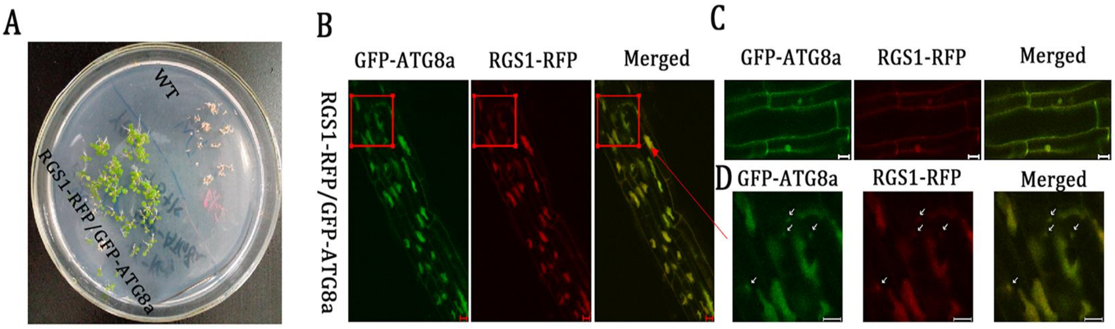

Figure S1. (A) RGS1-RFP/GFP-ATG8a were screened in Kan (100 μg/mL) and Basta (10 μg/mL) medium, and the WT was used as a control. **(B)** RFP-RGS1/GFP-ATG8a dark after starvation treatment for 24 h, 1 μM CA for 12 h, 6% glucose stress treatment for 30 min. **(C)** Normal seedlings. **(D)** an enlarged view of the red box mark. At least three independent experiments were performed. Scale bar =10 μm

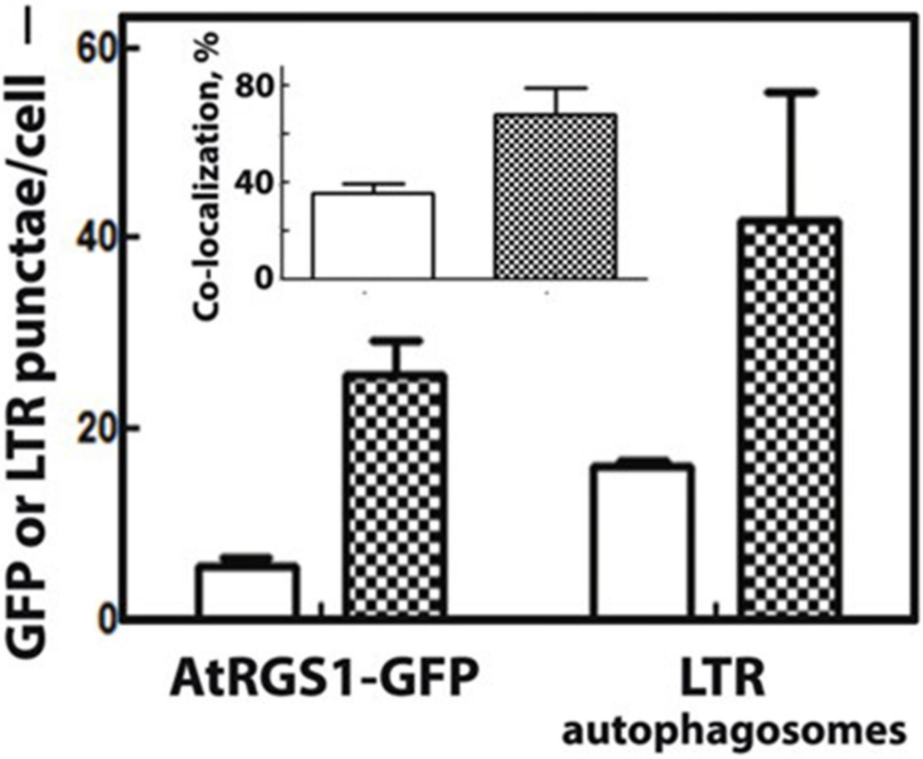

Figure S2. Quantification of (C) and (D) in Figure 4. The results showed that NtRGS1 and LTR co-localization of starvation is higher than that of normal growth conditions. (C) under MS 3% sucrose 5 days, and add 1 μM CA for 12 h (control). (D) RGS1 MS 3% sucrose 3 days, and starvation 2 days, and add 1 μM CA for 12 h (starvation).

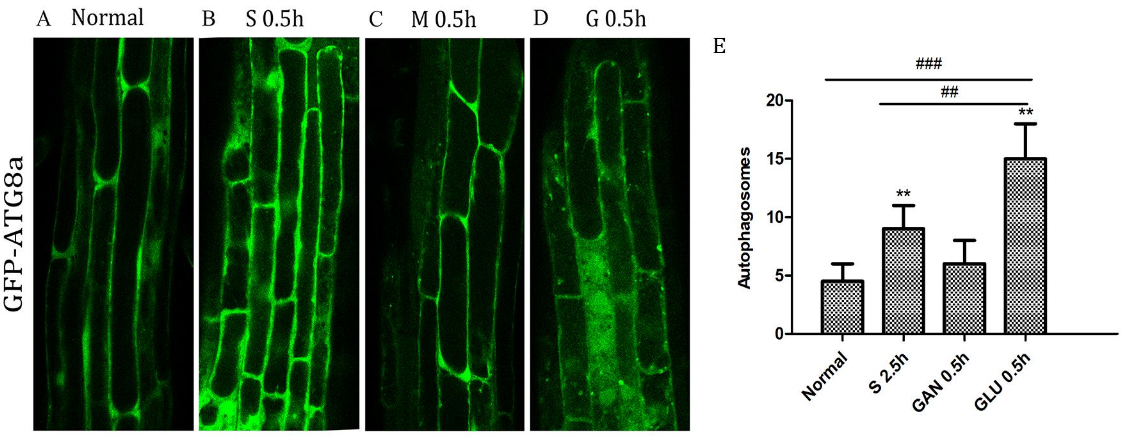

Figure S3. The observation utophagosomes. Autophagosomes labeled by GFP-ATG8a in roots of GFP-ATG8a plants with incubation in 1 μM CA for 12 h. **(A)** Normal seedlings. **(B)** Starved seedlings treatment for 2.5 h. **(C)** Mannitol seedlings treatment for 0.5 h. **(D)** Starved seedlings treatment for 2 h stimulated by 3% glucose treatment for 0.5 h. **(E)** Quantification of the GFP-ATG8a-labeled autophagosomes per cell. roots at the indicated times was used to calculate the autophagic activity. The mean and S.D. values were calculated from roots of six seedlings per time point. Results in three parallel experiments were used for the quantification. The asterisks indicate significant differences from the starved seedlings treated with glucose for 0 h. Scale bars =10 μm (***P* < 0.01; ##*P*<0.01 or ###*P*<0.001)

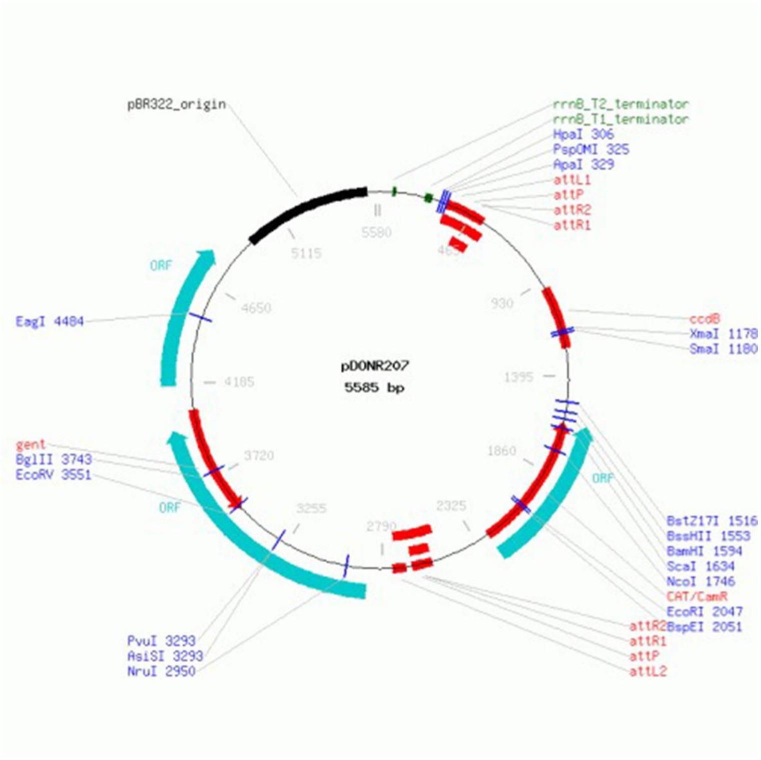

Figure S4. Vector pDONR207

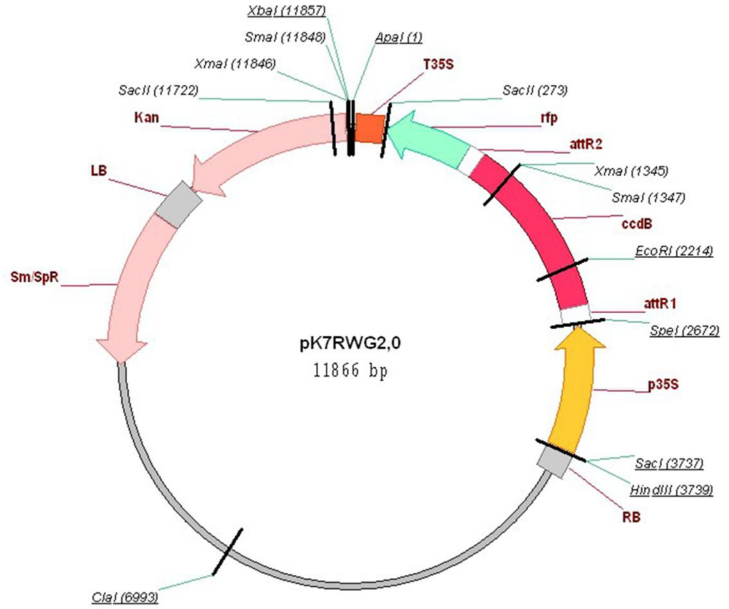

Figure S5. Vector pK7RWG2

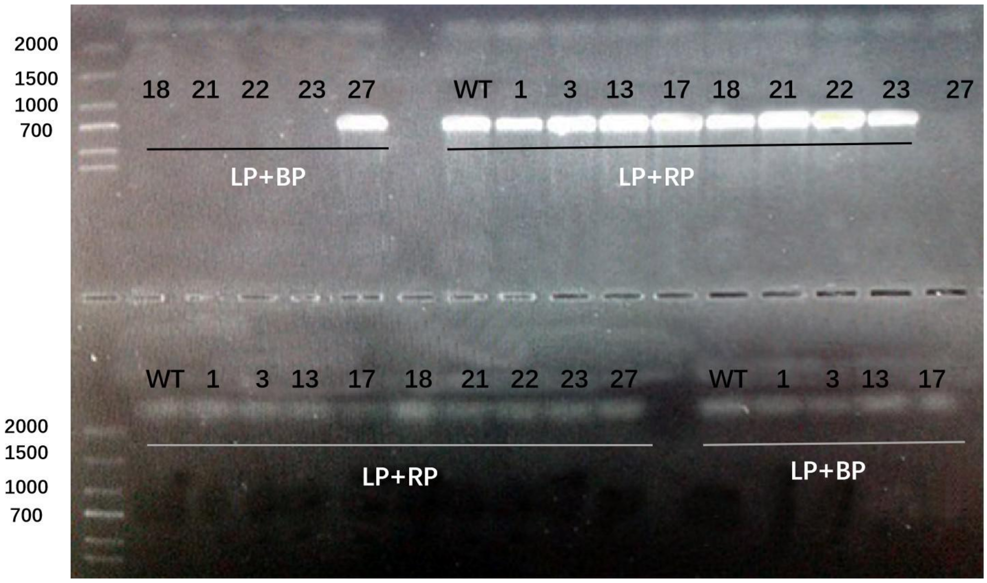

Figure S6. 1% agarose gel electrophoresis was observed and photographed using a gel imaging system. When the LP+BP or RP+BP primers were added, only one lane appeared as a homozygous mutant, as shown in Figure No. 27. The numbers are plant code, LP: insert left gene primer; RP: insert gene right primer; BP: insert gene primer. Maker: DL2000 Marker (TaKaRa)

rgs1 Primers

LP:TGTTGATGAAAAGCCTTAGCG
RP:TAGCTGCTACGCTGGAGAAAC

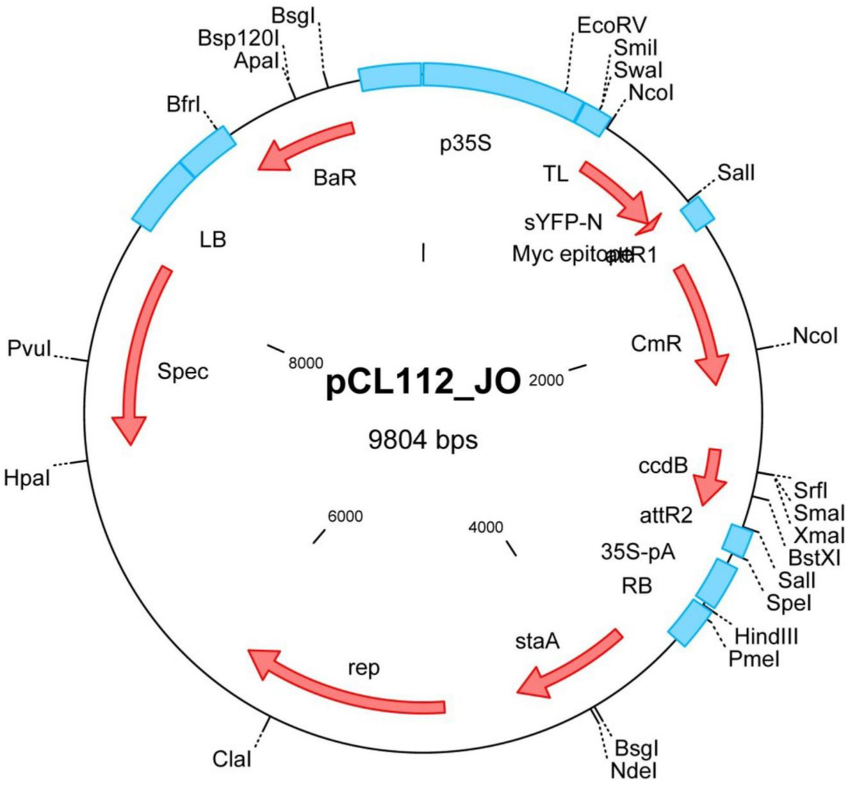

Figure S7. Vector pCL112_JO

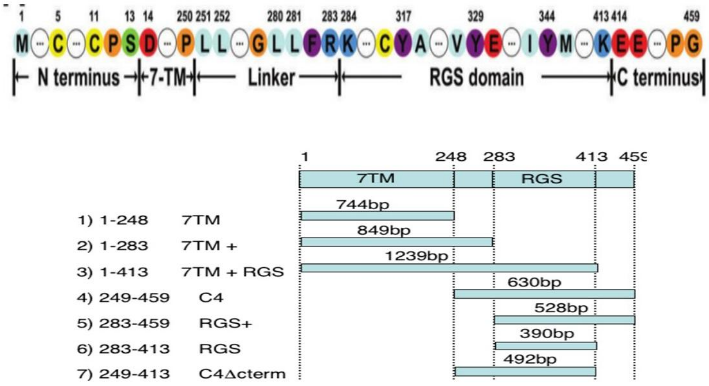

Figure S8. Domain structure of the RGS1 protein, NtRGS1 is expressed under 35S promoter in our constructs. The gene was cloned from BY-2 cDNA incl. 12 bp, of Kozak sequence in order to make the expression smoother. The following is the sequence of the construct

GGAGAATAAATTATGGCAGCTTGTGC
TAGCAAAGGAGGGTGTCCAAGCGACTATATGGCGCTTTCAATTGCCGTAGTGTCCATGATCTTACTTCTC
CTGAAAGCGA
CATTACCATACCTGATTCACAAGATTCCGAGGCCCAAAGGCAGTAGCTTTTGGCTTGTGGCAATTCAAG
TTCTAGCAAGC
TTCAATCTGTTGTTGTCAATTGTGATAGCTCTCAATTTTCTAAAGTTCAGAAAGAGACATTGGTGGAGGT
CTTGCTATCT
TTGGGCAGTCTGGATTGAAGGTCCACTCGGATTTGGTTTGCTGTTGAGCTGCCGTATTACACAGATATTC

CAACTATATT
ATATATTTGTGAAGAGACGTCTGCCACCAATTAGATCCTATATTTTTCTTTCGCTGATTCTCTTGCCGTGG
ATAGCTAGT
GCTGCCTTTATTCATATAAGAAAGCCTCTAAATTACCGATGTCACATGGGGACTGTGTGGATCATCCCTG
TTATGGGCCT
TCATGCATTATATGTTGTAGCTTTGATTGCTTTTACTGGGGCTGTGCGTCATGTGGAATTCAGATTTCATG
AACTCAAAG
ACCTTTGGAGAGGAATTCTTGTATCTGCAGCTTCCATTGGAATTTGGGTGGCTGCTTATGTTATGAATGA
AGTTCGCGAG
GATATATCATGGCTAGAAA
TTGCCTCTAGATTTTTGCTATTGGTTATGACAAGTGTCCTTGTGCTGGCATTCTTCTCTT
TCTCAATTTCTCAACCTCTTATCTCACTTATGAGCTTGAGGAAGAAGGATCATAAGGAGT
ACAAGACAATGAGCCAGGCATTAGGCATACATGATAGTGGGATTCATCTACAGAGGGAAC
CGATAAGCATTATAGATCCAGATGAACCTTTGGATAAGCTCCTGCTGAACCGAAGGTTCC
GGCAGTCCTTCATGGAATTTGCAGACAGTTGTTTGGCTGGGGAGAGTGTCCATTTCTATG
ATGAAGTGCAACAGTTTGATAAAATTCCTATTCAAGATCCAGTTAGGAGAATTTACATAG
CGCGCCACATAATAGAGAAGTATATAGCTGCAGGAGCACCAATGGAGGTGAACATTTCTC
ACAGAATTCGGCAGGAAATTTTGAGTACTAATGATCTCTCCCATACTAATCTCTTCAAAA
ATACTCTAGGTGAACTGATGCAGCTGATGAAACTGAATTTAGCAAAGGATTACTGGTCAT
CGATATATTTCATGAAGCTGAAAGACGAGGTCAGTATGAGAACAGTTGATCATGAGCTGG
AACATGCTAGCGGTTGGAACTTTTCTCCAAGATTGAGTTCTGTCCATTGTAGTGATGACC
CTTTTCAGCATGAACATAGCCCCAGGTATTCTGCTTGCCATAGTCATGATTCAAAACTGC
AATCTGAATTCGTCGACAAGCTTATGGTGAGCAAGGGCGAGGAGCTGTTCACCGGGGTGG
…GFP to be continued
Kozak sequence
ATG-NtRGS start
TCT-NtRGS stop
GFP
pENTR RGS pieces-BY-2
Nicotiana tabacum NtRGS1$^{1-248}$(7TM), NtRGS1$^{249-459}$(RGS domain), NtRGS1$^{249-413}$ (RGS domain not included C terminus)

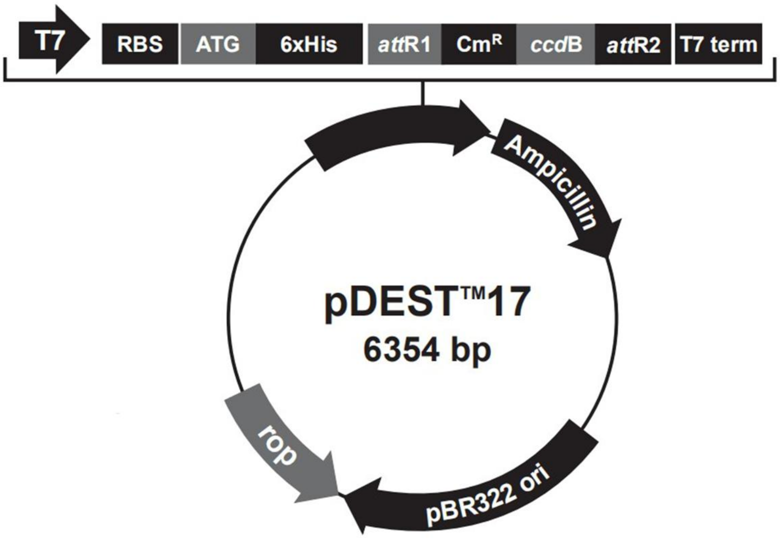

Figure S9. Vector pDEST17

SVideo.
