# Supplementary figures and images for: Correlation of Autophagosome Formation with Degradation and Endocytosis Arabidopsis Regulator of G-Protein Signaling (RGS1) through ATG8a
Source: Int J Mol Sci. 2019 Aug 27;20(17):4190. doi: 10.3390/ijms20174190 (PMC6747245; doi:10.3390/ijms20174190)

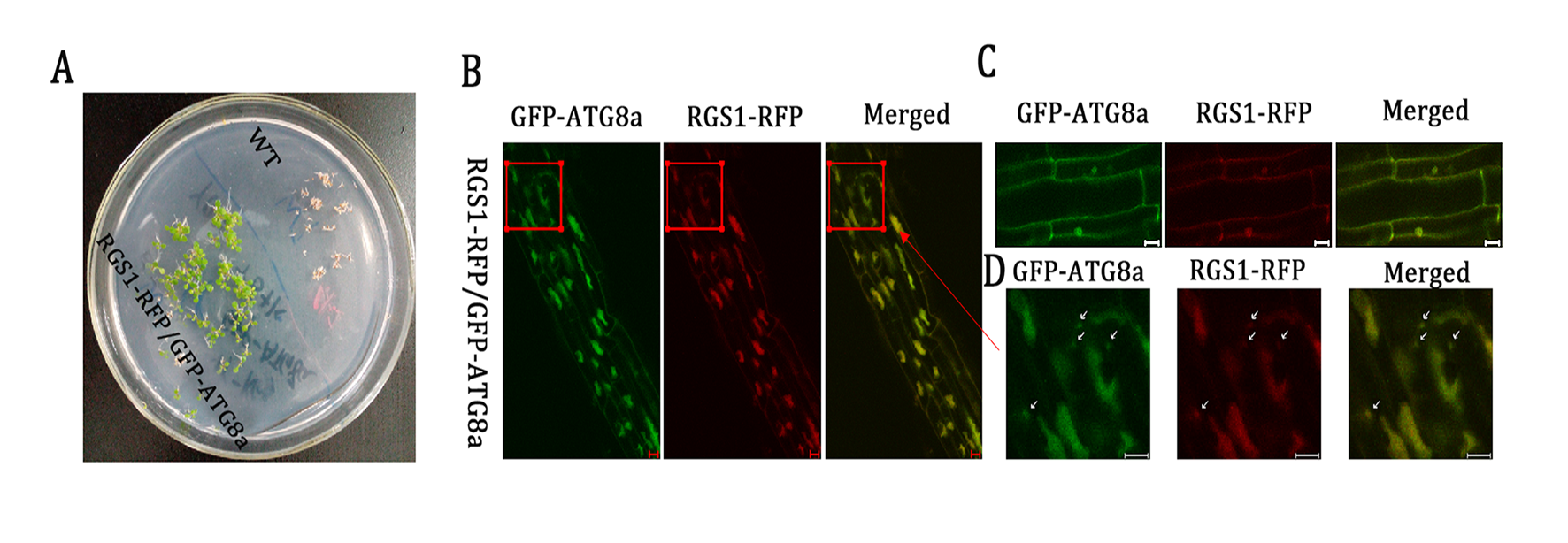

Supplement: Supplementary file 1 [file ijms-20-04190-s001.zip › supplementary figure/Figure S1.tif]

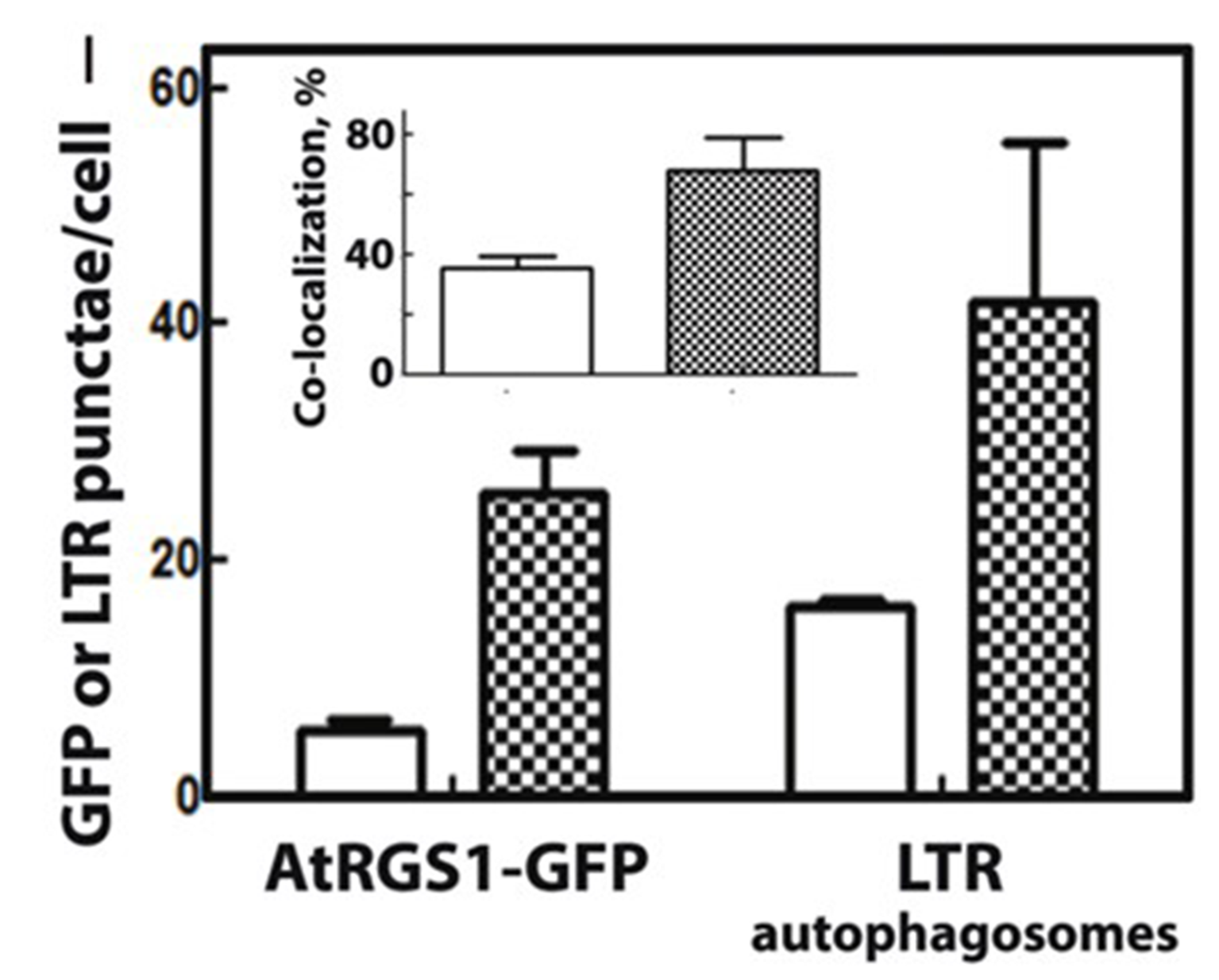

Supplement: Supplementary file 1 [file ijms-20-04190-s001.zip › supplementary figure/Figure S2.tif]

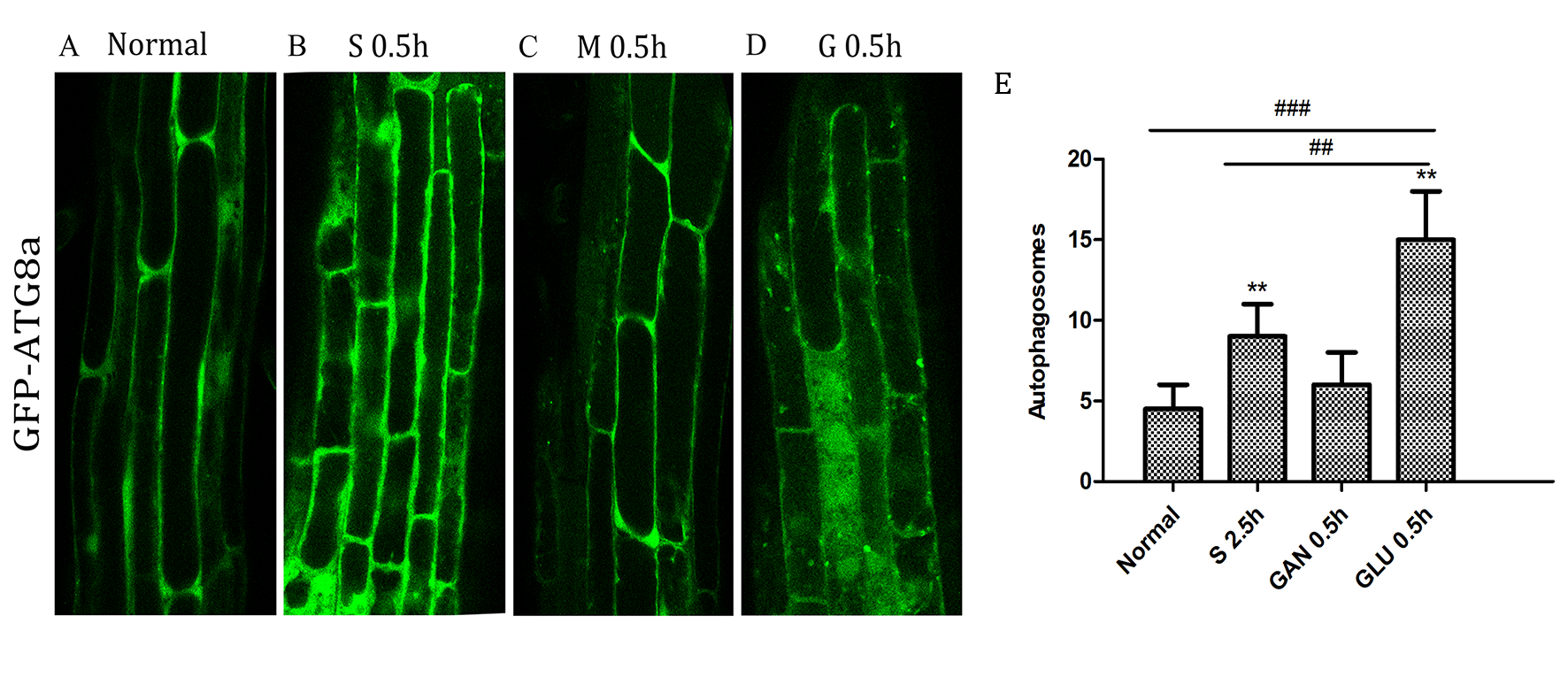

Supplement: Supplementary file 1 [file ijms-20-04190-s001.zip › supplementary figure/Figure S3.tif]

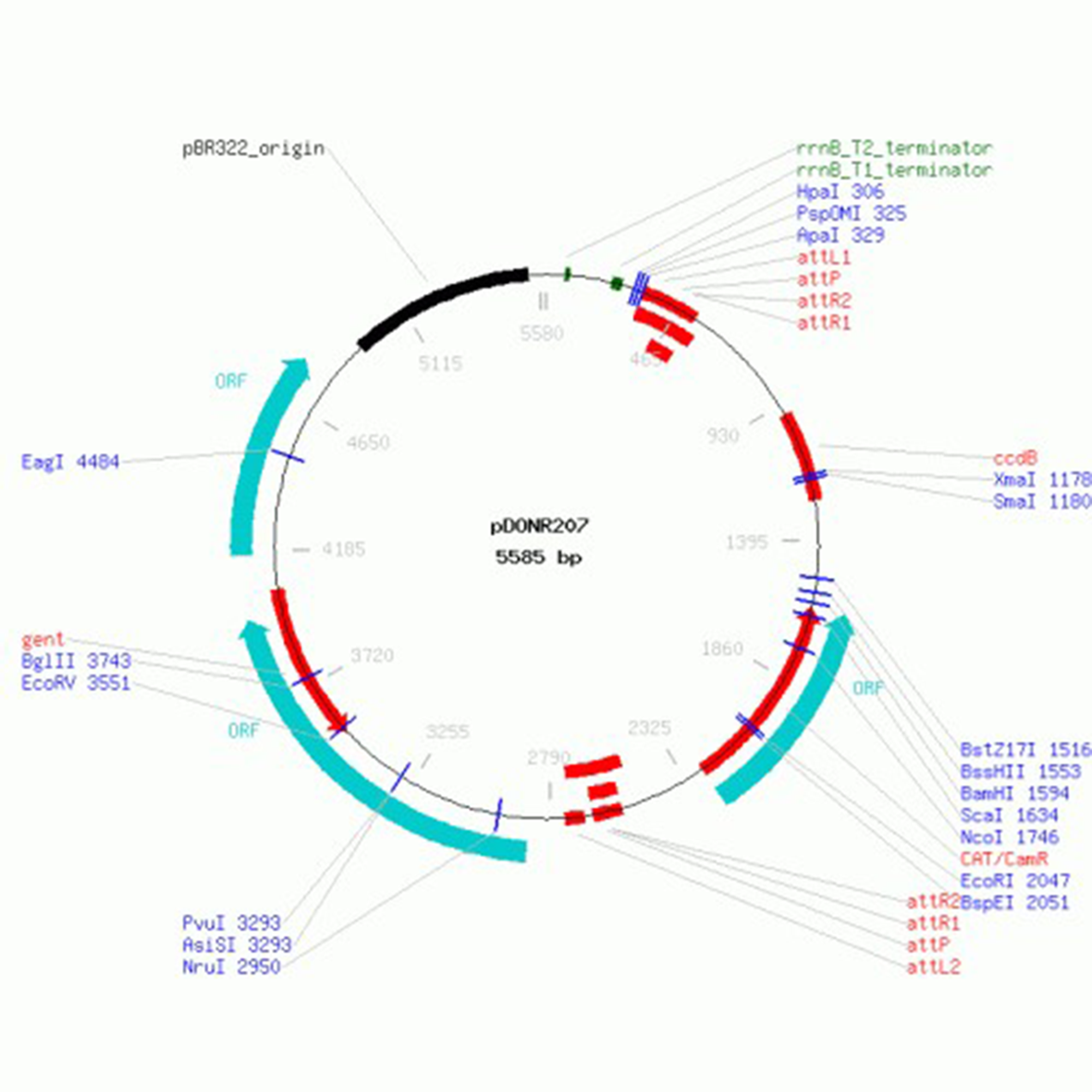

Supplement: Supplementary file 1 [file ijms-20-04190-s001.zip › supplementary figure/Figure S4.tif]

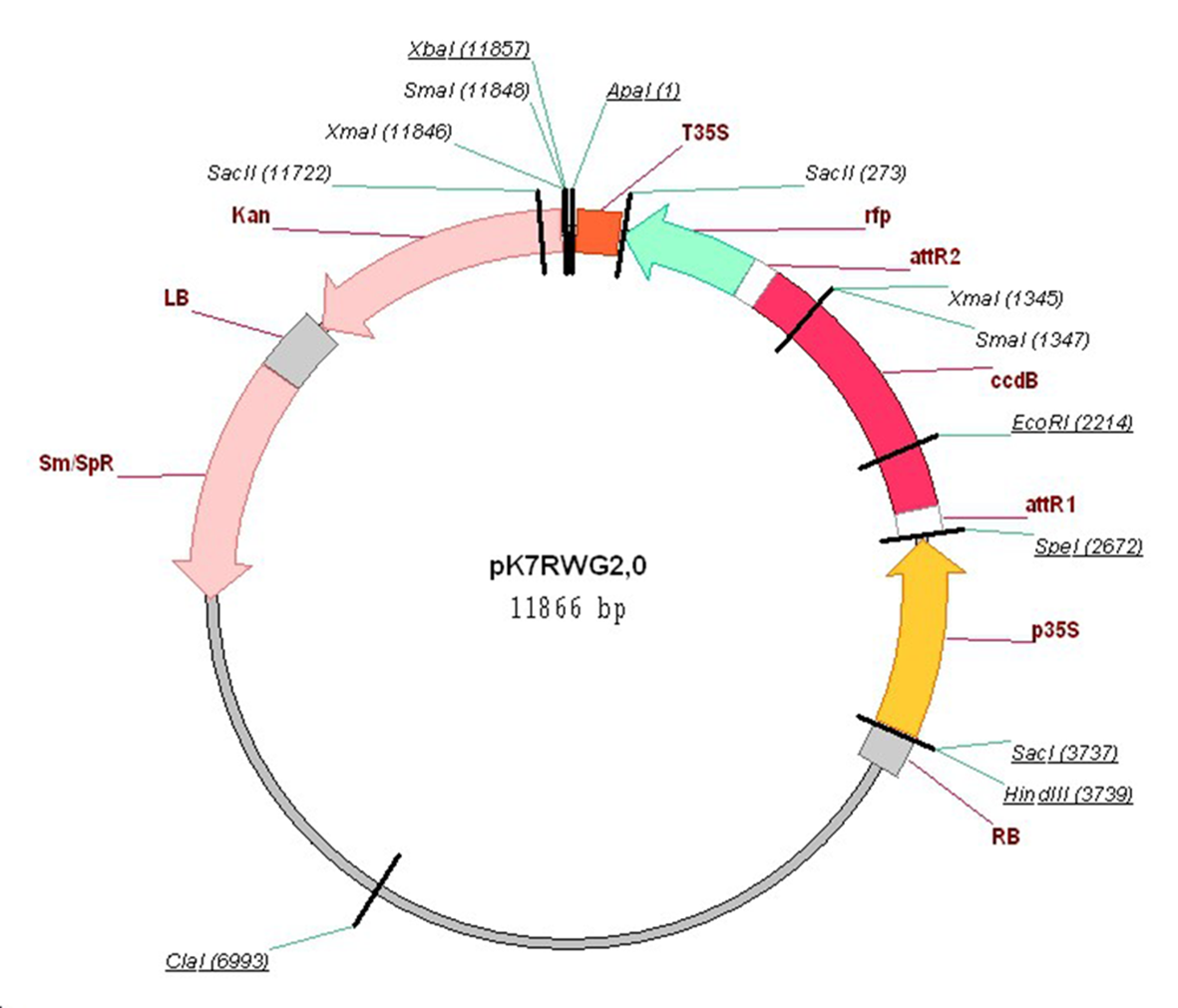

Supplement: Supplementary file 1 [file ijms-20-04190-s001.zip › supplementary figure/Figure S5.tif]

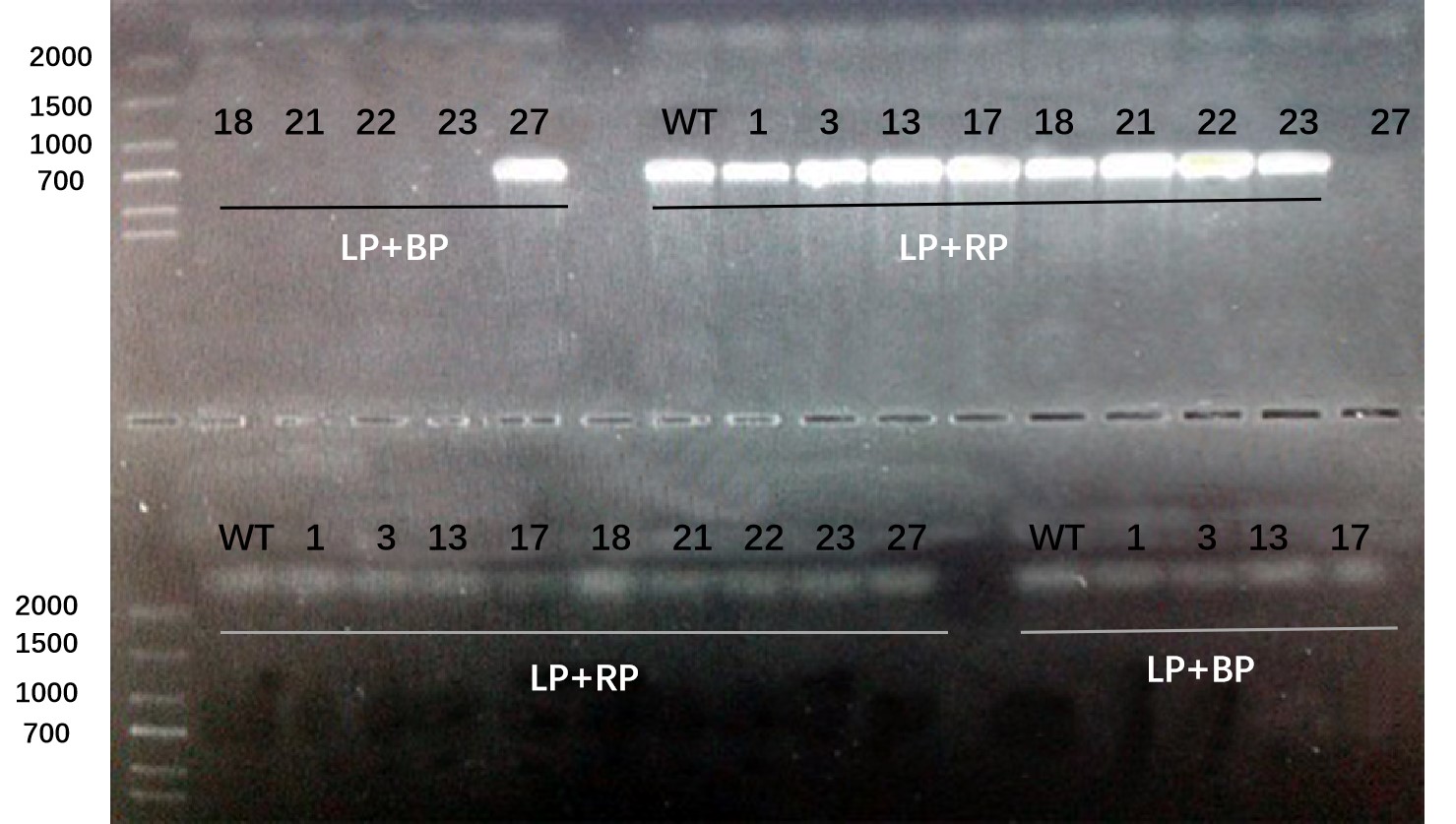

Supplement: Supplementary file 1 [file ijms-20-04190-s001.zip › supplementary figure/Figure S6.jpg]

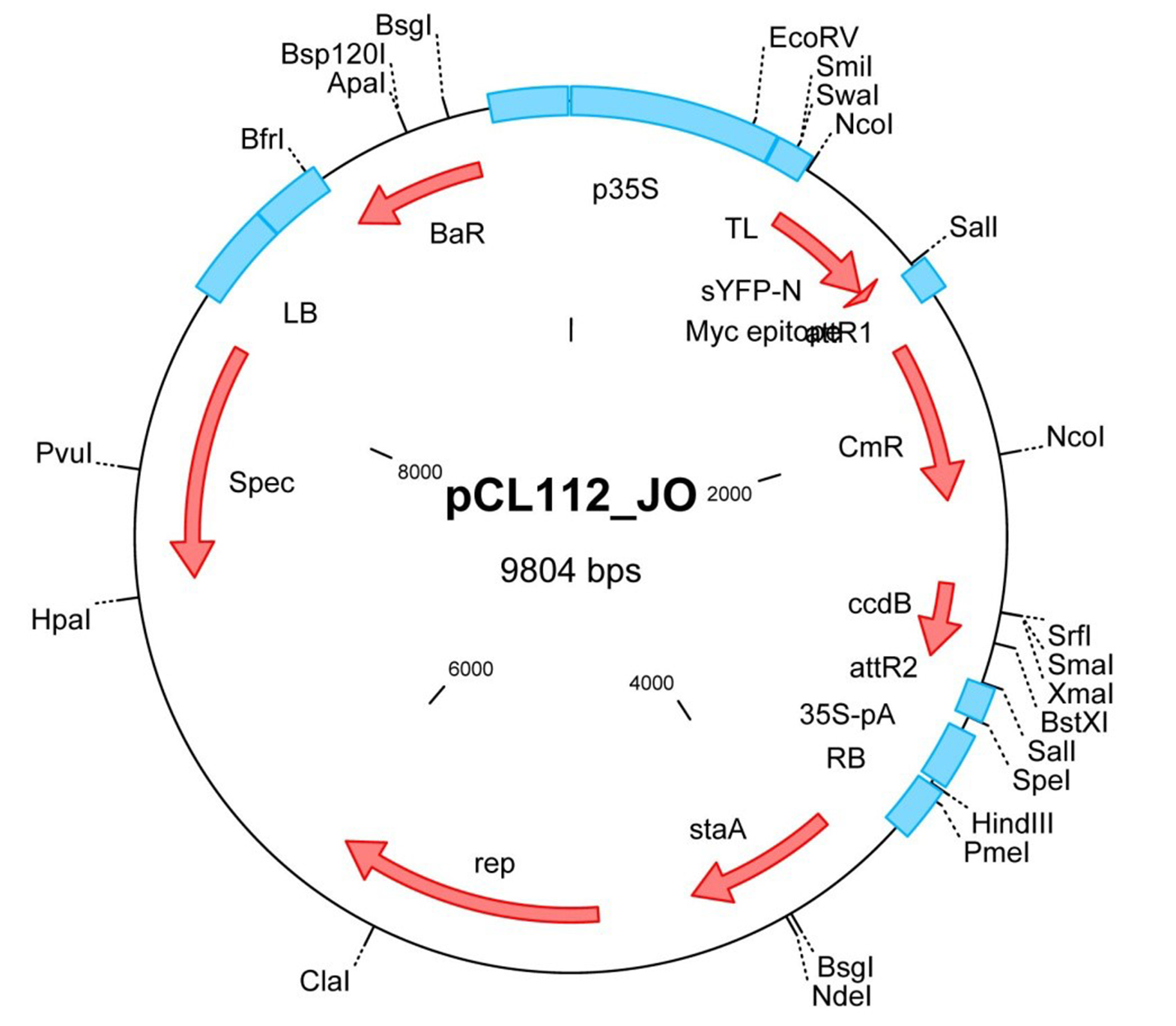

Supplement: Supplementary file 1 [file ijms-20-04190-s001.zip › supplementary figure/Figure S7.tif]

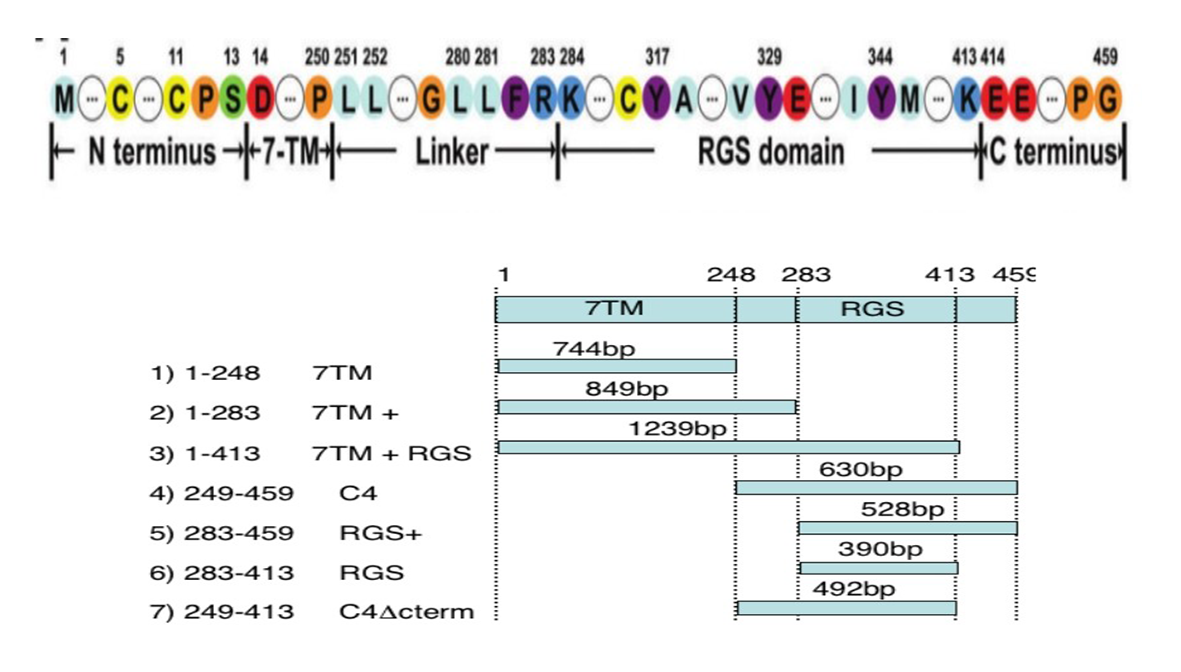

Supplement: Supplementary file 1 [file ijms-20-04190-s001.zip › supplementary figure/Figure S8.tif]

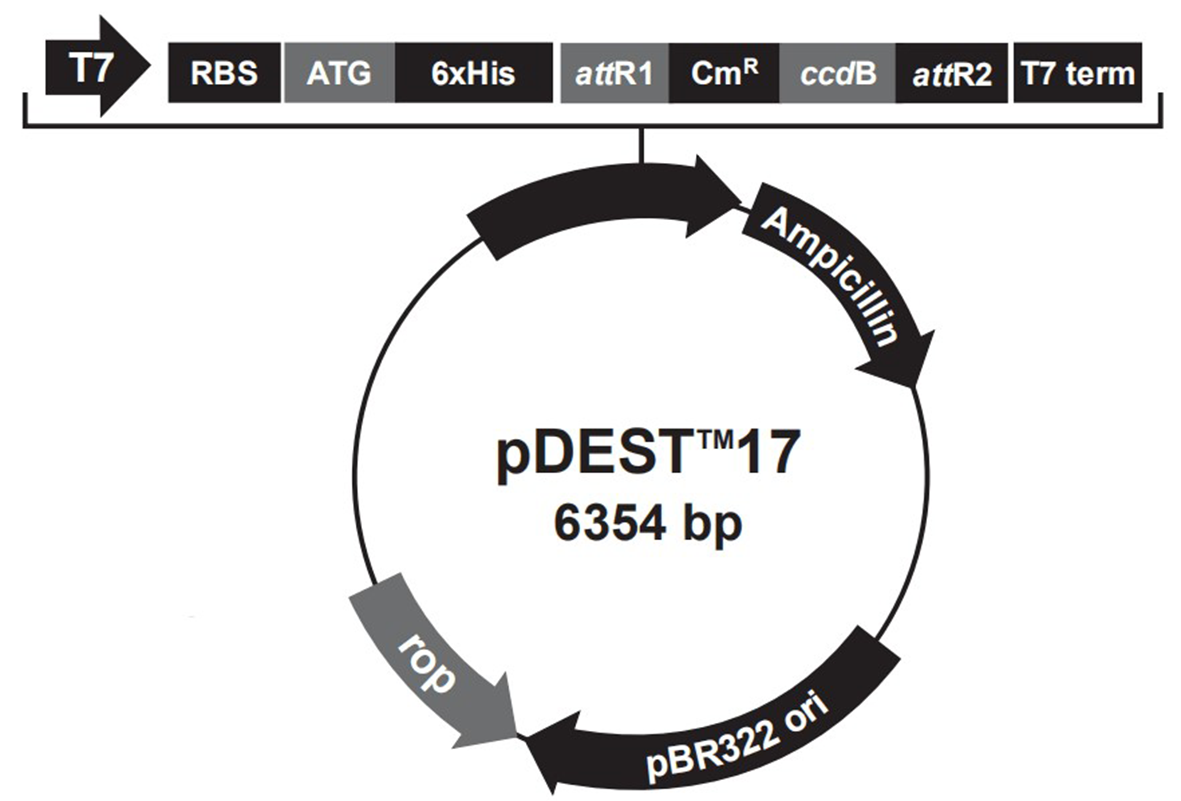

Supplement: Supplementary file 1 [file ijms-20-04190-s001.zip › supplementary figure/Figure S9.tif]
