# Supplementary material for: Correlation of Autophagosome Formation with Degradation and Endocytosis Arabidopsis Regulator of G-Protein Signaling (RGS1) through ATG8a
Source: Int J Mol Sci. 2019 Aug 27;20(17):4190. doi: 10.3390/ijms20174190 (PMC6747245; doi:10.3390/ijms20174190)
Supplement: Supplementary file 1 [file ijms-20-04190-s001.zip › manuscript supplementary.doc]

**Supplementary data**

**Figure S1. (A)** RGS1-RFP/GFP-ATG8a were screened in Kan (100 µg/mL) and Basta (10 µg/mL) medium, and the WT was used as a control. **(B)** RFP-RGS1/GFP-ATG8a dark after starvation treatment for 24 h, 1 µM CA for 12 h, 6% glucose stress treatment for 30 min. **(C)** Normal seedlings. **(D)** an enlarged view of the red box mark. At least three independent experiments were performed. Scale bar =10 µm

**Figure S2.** Quantification of (C) and (D) in Figure 4. The results showed that NtRGS1 and LTR co-localization of starvation is higher than that of normal growth conditions. (C) under MS 3% sucrose 5 days, and add 1 µM CA for 12 h (control). (D) RGS1 MS 3% sucrose 3 days, and starvation 2 days, and add 1 µM CA for 12 h (starvation).

**Figure S3.** The observation utophagosomes. Autophagosomes labeled by GFP-ATG8a in roots of GFP-ATG8a plants with incubation in 1 µM CA for 12 h. **(A)** Normal seedlings. **(B)** Starved seedlings treatment for 2.5 h. **(C)** Mannitol seedlings treatment for 0.5 h. **(D)** Starved seedlings treatment for 2 h stimulated by 3% glucose treatment for 0.5 h. **(E)** Quantification of the GFP-ATG8a-labeled autophagosomes per cell. roots at the indicated times was used to calculate the autophagic activity. The mean and S.D. values were calculated from roots of six seedlings per time point. Results in three parallel experiments were used for the quantification. The asterisks indicate significant differences from the starved seedlings treated with glucose for 0 h. Scale bars =10 µm (***P* < 0.01; ^##^*P*<0.01 or ^###^*P*<0.001)

**Figure S4.** Vector pDONR207

**Figure S5.** Vector pK7RWG2

**Figure S6.** 1% agarose gel electrophoresis was observed and photographed using a gel imaging system. When the LP+BP or RP+BP primers were added, only one lane appeared as a homozygous mutant, as shown in Figure No. 27. The numbers are plant code, LP: insert left gene primer; RP: insert gene right primer; BP: insert gene primer. Maker: DL2000 Marker (TaKaRa)

*rgs1* Primers

LP:TGTTGATGAAAAGCCTTAGCG

RP:TAGCTGCTACGCTGGAGAAAC

**Figure S7.** Vector pCL112_JO

**Figure S8.** Domain structure of the RGS1 protein, NtRGS1 is expressed under 35S promoter in our constructs. The gene was cloned from BY-2 cDNA incl. 12 bp, of Kozak sequence in order to make the expression smoother. The following is the sequence of the construct

GGAGAATAAATTATGGCAGCTTGTGC

TAGCAAAGGAGGGTGTCCAAGCGACTATATGGCGCTTTCAATTGCCGTAGTGTCCATGATCTTACTTCTCCTGAAAGCGA

CATTACCATACCTGATTCACAAGATTCCGAGGCCCAAAGGCAGTAGCTTTTGGCTTGTGGCAATTCAAGTTCTAGCAAGC

TTCAATCTGTTGTTGTCAATTGTGATAGCTCTCAATTTTCTAAAGTTCAGAAAGAGACATTGGTGGAGGTCTTGCTATCT

TTGGGCAGTCTGGATTGAAGGTCCACTCGGATTTGGTTTGCTGTTGAGCTGCCGTATTACACAGATATTCCAACTATATT

ATATATTTGTGAAGAGACGTCTGCCACCAATTAGATCCTATATTTTTCTTTCGCTGATTCTCTTGCCGTGGATAGCTAGT

GCTGCCTTTATTCATATAAGAAAGCCTCTAAATTACCGATGTCACATGGGGACTGTGTGGATCATCCCTGTTATGGGCCT

TCATGCATTATATGTTGTAGCTTTGATTGCTTTTACTGGGGCTGTGCGTCATGTGGAATTCAGATTTCATGAACTCAAAG

ACCTTTGGAGAGGAATTCTTGTATCTGCAGCTTCCATTGGAATTTGGGTGGCTGCTTATGTTATGAATGAAGTTCGCGAG

GATATATCATGGCTAGAAA

TTGCCTCTAGATTTTTGCTATTGGTTATGACAAGTGTCCTTGTGCTGGCATTCTTCTCTT

TCTCAATTTCTCAACCTCTTATCTCACTTATGAGCTTGAGGAAGAAGGATCATAAGGAGT

ACAAGACAATGAGCCAGGCATTAGGCATACATGATAGTGGGATTCATCTACAGAGGGAAC

CGATAAGCATTATAGATCCAGATGAACCTTTGGATAAGCTCCTGCTGAACCGAAGGTTCC

GGCAGTCCTTCATGGAATTTGCAGACAGTTGTTTGGCTGGGGAGAGTGTCCATTTCTATG

ATGAAGTGCAACAGTTTGATAAAATTCCTATTCAAGATCCAGTTAGGAGAATTTACATAG

CGCGCCACATAATAGAGAAGTATATAGCTGCAGGAGCACCAATGGAGGTGAACATTTCTC

ACAGAATTCGGCAGGAAATTTTGAGTACTAATGATCTCTCCCATACTAATCTCTTCAAAA

ATACTCTAGGTGAACTGATGCAGCTGATGAAACTGAATTTAGCAAAGGATTACTGGTCAT

CGATATATTTCATGAAGCTGAAAGACGAGGTCAGTATGAGAACAGTTGATCATGAGCTGG

AACATGCTAGCGGTTGGAACTTTTCTCCAAGATTGAGTTCTGTCCATTGTAGTGATGACC

CTTTTCAGCATGAACATAGCCCCAGGTATTCTGCTTGCCATAGTCATGATTCAAAACTGC

AATCTGAATTCGTCGACAAGCTTATGGTGAGCAAGGGCGAGGAGCTGTTCACCGGGGTGG

…GFP to be continued

Kozak sequence

ATG-NtRGS start

TCT-NtRGS stop

GFP

pENTR RGS pieces-BY-2

*Nicotiana tabacum NtRGS1*^1-248^(7TM), NtRGS1^249-459^(RGS domain), NtRGS1^249-413^ (RGS domain not included C terminus)

**Figure S9.** Vector pDEST17

**SVideo.**
